# Cancer Information Burden Among US Cancer Survivors Using Health Information National Trends Survey 2022 and 2024: Cross-Sectional Study

**DOI:** 10.2196/101310

**Published:** 2026-10-28

**Authors:** Jingjing Gao, Sharon V Munroe, Yue Zhang, Jason H Windett

**Affiliations:** 1 Department of Management, Policy & Community Health, UTHealth Houston School of Public Health, 1200 Pressler Street, Houston, TX, 77030, United States, 980 213 6680; 2 Department of Health and Human Performance, College of Health Professions, University of Texas Rio Grande Valley, Houston, TX, United States; 3 Department of Biostatistics and Data Science, School of Public Health, University of Texas Health Science Center at Houston, Houston, TX, United States; 4 School of Data Science, University of North Carolina at Charlotte, Charlotte, NC, United States

**Keywords:** cancer survivors, cancer information burden, digital health, health literacy, generative AI

## Abstract

**Background:**

Digital health platforms have become increasingly important sources of health information for cancer survivors. Cancer survivors frequently rely on digital resources to obtain information related to diagnosis, treatment, survivorship, and follow-up care. In this study, cancer information burden is defined as the perceived challenges associated with seeking, understanding, and evaluating cancer-related information, including the effort required to obtain information, frustration during information seeking, difficulty understanding information, and concern regarding information quality. As the online health information environment becomes increasingly complex, some survivors may experience difficulty locating, evaluating, and understanding cancer-related information. However, limited nationally representative research has examined how cancer information burden differs across demographic and socioeconomic groups.

**Objective:**

This study examined cancer information burden and associated demographic and socioeconomic factors among US cancer survivors using nationally representative data from the Health Information National Trends Survey (HINTS) 2022 and 2024.

**Methods:**

Data were drawn from pooled cycles of the HINTS conducted in 2022 and 2024. Survey-weighted regression analyses were used to evaluate factors associated with cancer information burden among cancer survivors. Outcomes included the perceived effort required to obtain cancer information, frustration during information seeking, difficulty understanding cancer information, and concerns regarding information quality. Composite measures of cancer information burden were also examined. Models were adjusted for demographic and socioeconomic characteristics, including age, sex, race and ethnicity, educational attainment, household income, geographic region, and survey cycle.

**Results:**

Hispanic cancer survivors reported a significantly greater cancer information burden, including higher levels of perceived effort and frustration associated with obtaining and understanding cancer-related information, compared with non-Hispanic White survivors. Non-Hispanic Asian survivors also demonstrated elevated levels of information burden, although estimates were less precise because of smaller subgroup sample sizes. Postbaccalaureate education was associated with lower frustration during cancer information seeking. Female survivors reported lower levels of difficulty understanding cancer-related information and lower overall information burden than male survivors. Differences between the 2022 and 2024 survey cycles were not statistically significant after adjustment.

**Conclusions:**

Differences in cancer information burden persist among US cancer survivors. Hispanic and non-Hispanic Asian survivors, and individuals with lower educational attainment, may experience greater challenges obtaining and navigating cancer-related information. These findings support the development of accessible, culturally responsive, and health literacy–sensitive cancer communication strategies.

## Introduction

Cancer survivors are increasingly turning to online health resources to manage diagnosis, treatment, survivorship, symptoms, and long-term care. Digital health tools like patient portals, educational websites, telehealth services, and search engines have become essential parts of today’s cancer information landscape [1-3]. As health care systems expand their digital communication infrastructure, survivors are likely to encounter more complex online information environments. Emerging technologies, including conversational agents and algorithmically supported search tools, represent 1 component of the changing digital information environment [4,5]. However, the present study did not assess awareness, use, or exposure to these technologies.

Cancer survivors may be especially vulnerable to these changes because cancer-related information is often medically complex, emotionally distressing, and hard to interpret. Previous studies have shown that survivors commonly face information overload, frustration, and uncertainty when looking for cancer-related information online [6-11]. In this study, cancer information burden is defined as the perceived difficulty associated with seeking, understanding, and evaluating cancer-related information, including effort, frustration, difficulty understanding, and concern regarding information quality. Differences in digital access, education levels, health literacy, and trust in health information sources can further affect individuals’ capacity to take advantage of changing digital health environments [12-14].

Prior studies have documented demographic and socioeconomic differences in cancer information seeking, information-source preferences, patient-centered communication, and digital information use among cancer survivors. Earlier Health Information National Trends Survey (HINTS)–based and population-based studies found that age, race and ethnicity, educational attainment, income, cancer history, and perceived health were associated with information-seeking behaviors and experiences [15-18]. These studies establish that cancer information experiences are not uniform across survivor populations. However, less is known about whether these patterns remain evident in the most recent HINTS cycles and how multiple dimensions of perceived information-seeking burden perform when examined separately and as a validated composite measure. Older, Black, and Latino seniors, and seniors from other minority groups have less access to digital tools, less online experience, and are less confident using web-based health resources, making them less likely to use eHealth or mobile health tools [19-21]. Nonetheless, limited nationally representative research has examined cancer information burden among survivors during the 2022 and 2024 period or identified the population groups most likely to report difficulty obtaining and understanding cancer information. Identifying population groups that report greater difficulty accessing and understanding cancer information may inform the development of more accessible digital communication strategies. It may also provide a baseline for future studies that directly assess how emerging digital technologies affect information access and navigation.

Using pooled HINTS 2022 and 2024 data, this study provides an updated, nationally representative assessment of cancer information-seeking burden among US cancer survivors. The analysis examined 4 related but distinct experiences: effort in obtaining information, frustration during information seeking, difficulty understanding information, and concern regarding information quality. We also evaluated the internal consistency and dimensional structure of a composite burden measure and examined its demographic and socioeconomic correlates. The study was intended to update and extend prior evidence rather than attribute observed patterns to emerging technologies. The primary research question was as follows: which demographic and socioeconomic characteristics are associated with perceived effort, frustration, difficulty understanding, concern regarding information quality, and overall cancer information-seeking burden among US cancer survivors? As a secondary analysis, we asked the following: does the association between age and cancer information-seeking burden differ between HINTS 2022 and HINTS 2024? Age was selected because older and younger cancer survivors may differ in digital access, familiarity with online health information, and experience navigating digital health resources.

This study was informed by the Comprehensive Model of Information Seeking, which proposes that health information-seeking experiences are shaped by antecedent characteristics, including demographic factors, personal experience, salience, and beliefs, as well as characteristics of information channels and sources [22-25]. In the context of cancer survivorship, age, sex, race and ethnicity, educational attainment, household income, geographic context, and prior cancer-related experience may influence how individuals seek, understand, and evaluate information. The present analysis focused on demographic and socioeconomic antecedents available consistently in HINTS 2022 and 2024. Because the dataset did not include all constructs required for a complete test of the framework, including detailed cancer characteristics, digital access, eHealth literacy, prior health care experiences, information-source characteristics, and trust, the framework was used to guide interpretation rather than to test the full model.

## Methods

### Data Source and Study Population

This study used pooled cross-sectional data from the 2022 and 2024 cycles of the HINTS, a nationally representative survey conducted by the National Cancer Institute that explores health communication behaviors, health information seeking, and digital health engagement among US adults. These were the 2 most recent publicly available nationally representative HINTS datasets at the time of analysis. A standard HINTS survey cycle was not conducted in 2023; therefore, no nationally representative HINTS 2023 dataset was available for inclusion. Cancer survivors were identified using the HINTS question that asked whether a doctor or other health professional had ever told the respondent that they had cancer. Respondents who answered “yes” were classified as cancer survivors. This definition captured a self-reported cancer diagnosis at any time and was not restricted to diagnoses occurring within the previous year. Respondents who answered “no” were excluded from the cancer-survivor sample. No additional cancer-specific eligibility criteria were applied based on cancer type, stage, treatment status, recurrence, or time since diagnosis. Pooling the 2 survey cycles yielded 1972 cancer survivors. For outcome-specific descriptive analyses, respondents were required to have a valid response to the corresponding cancer information burden item. The composite score was calculated as the mean of available responses across the 4 burden items. Fully adjusted regression models were further restricted to respondents with complete data for the relevant outcome and all included covariates. Survey cycle was not treated as a measure or proxy of generative AI (GenAI) exposure because HINTS did not assess respondents’ awareness, use, or exposure to GenAI tools.

### Measures

#### Cancer Information Burden Outcomes

Four HINTS items were used to assess cancer information-seeking burden, defined as the perceived effort, frustration, difficulty understanding, and concern regarding information quality experienced during the search for cancer-related information. Respondents were asked, “based on the results of your most recent search for information about cancer, how much do you agree or disagree with each of the following statements?” The statements were as follows: “it took a lot of effort to get the information you needed,” “you felt frustrated during your search for the information,” “you were concerned about the quality of the information,” and “the information you found was hard to understand.” These items represent perceived effort, frustration, concern regarding information quality, and difficulty understanding cancer information, respectively. The original HINTS response options were strongly disagree, somewhat disagree, somewhat agree, and strongly agree.

Each item was coded on a 4-point ordinal scale ranging from 0 (strongly disagree) to 3 (strongly agree), with higher values indicating greater perceived cancer information burden. Responses that were coded as inapplicable, filter missing, not ascertained, or answered in error in the original HINTS data were treated as missing. The 4 items were analyzed separately as ordinal outcomes.

An overall cancer information burden score was calculated as the arithmetic mean of the available responses across the 4 coded burden items. The resulting score ranged from 0 to 3, with higher scores indicating greater overall perceived cancer information burden.

The 4-item composite demonstrated high internal consistency, with a Cronbach α of 0.881. Exploratory factor analysis supported a single-factor structure. The first factor had an eigenvalue of 2.948 and explained 73.7% of the total variance. Factor loadings were 0.876 for perceived effort, 0.898 for frustration, 0.820 for concern regarding information quality, and 0.838 for difficulty understanding. These findings support combining the 4 items into a composite score while retaining the individual items for dimension-specific analyses.

#### Covariates

Covariates were selected using a theory-informed approach based on the Comprehensive Model of Information Seeking and prior cancer communication literature. Demographic and socioeconomic variables were treated as antecedent characteristics that may shape cancer information-seeking experiences. Demographic and socioeconomic covariates included age, sex, race and ethnicity, educational attainment, household income, geographic census division, and survey cycle. Race and ethnicity categories included non-Hispanic White, non-Hispanic Black, Hispanic, non-Hispanic Asian, and non-Hispanic Other populations. Educational attainment was categorized as less than high school, high school graduate, some college, bachelor’s degree, and postbaccalaureate degree. Household income was categorized into 6 groups ranging from less than US $20,000 annually to US $100,000 or greater. Geographic variation was assessed using US census divisions. Survey cycle was included as a temporal covariate to evaluate whether cancer information burden differed between HINTS 2022 and HINTS 2024; it was not interpreted as a proxy for GenAI exposure.

#### Missing Data and Analytic Samples

Of the 1972 cancer survivors in the pooled HINTS 2022 and 2024 samples, the number of respondents with valid data differed across the cancer information burden outcomes. Valid outcome data were available for 1373 respondents for perceived effort in obtaining cancer information, 1366 for difficulty understanding cancer information, 1367 for frustration during information seeking, and 1368 for concern regarding information quality. The composite cancer information burden score was available for 1389 respondents because it was calculated as the mean of available responses to the 4 burden items.

Adjusted regression models used complete-case estimation. Respondents were included in a given model only when they had valid data for the corresponding outcome and all covariates included in that model. Therefore, respondents with valid outcome data could contribute to descriptive analyses but were excluded from adjusted analyses when age, sex, race and ethnicity, education, household income, census division, or survey-cycle information were missing. Household income had the largest amount of missing covariate data and accounted for much of the reduction in analytic sample size.

The final complete-case regression samples included 957 respondents for perceived effort, 952 for difficulty understanding, 953 for frustration, 956 for concern regarding information quality, and 969 for the composite score. No values were imputed in the primary analyses.

### Statistical Analysis

Survey-weighted analyses were conducted to account for the complex HINTS sampling design, including stratification, clustering, and sampling weights. Covariates were selected using the Comprehensive Model of Information Seeking and prior cancer communication literature. Age, sex, race and ethnicity, educational attainment, household income, US census division, and survey cycle were included as antecedent demographic and socioeconomic characteristics potentially associated with cancer information-seeking burden. Missing data were handled using complete-case analysis. Each regression model included only respondents with complete data for the outcome of interest and all covariates. Consequently, analytic sample sizes varied slightly across outcomes because of differences in item nonresponse. No imputation procedures were performed.

Survey-weighted proportional-odds ordinal logistic regression models were estimated for the 4 ordinal cancer information burden outcomes. Results are presented as odds ratios (ORs) with 95% CIs. An OR greater than 1 indicates higher odds of reporting a greater level of cancer information burden, whereas an OR below 1 indicates lower odds.

The proportional-odds assumption was assessed for each ordinal outcome using parallel-lines diagnostic tests. Evidence of nonproportionality was identified for perceived effort in obtaining cancer information and concern regarding information quality, while findings for difficulty understanding cancer information were mixed across tests. No evidence of nonproportionality was identified for frustration during information seeking. For outcomes showing evidence of assumption violations, generalized ordered logistic regression models with partial proportional odds were estimated as sensitivity analyses, allowing selected coefficients to vary across outcome thresholds. These sensitivity analyses yielded conclusions that were substantively consistent with the primary models.

Survey-weighted linear regression was used to examine the composite cancer information burden score. Cronbach α and exploratory factor analysis were used to assess the internal consistency and dimensional structure of the 4-item composite measure.

As a secondary analysis, an age × survey cycle interaction term was added to each model to assess whether age-related associations with cancer information burden differed between HINTS 2022 and HINTS 2024. Age was selected as the interaction of interest because it may be associated with access to, familiarity with, and navigation of digital health information environments. Additional interactions were not systematically examined because the available sample size limited statistical power for multiple subgroup analyses, and extensive interaction testing could increase the likelihood of chance findings. Statistical significance was assessed using 2-sided tests with an α level of .05. All analyses were conducted using Stata (version 19.5; StataCorp LLC).

### Ethical Considerations

This study was a secondary analysis of publicly available, deidentified data from the National Cancer Institute’s HINTS. Because the dataset is publicly available and contains no identifiable participant information, a separate institutional review board review was not required for this study. The original HINTS data collection protocols were reviewed and approved by the Westat Institutional Review Board, and all participants provided informed consent before participating.

## Results

### Sample Characteristics

Figure 1 presents the study population selection process, while Table 1 summarizes the weighted descriptive characteristics of US cancer survivors included in the analytic sample. The pooled analytic sample included 1972 US cancer survivors from the 2022 and 2024 HINTS cycles. The unweighted sample included 900 (45.6%) respondents from HINTS 2022 and 1072 (54.4%) from HINTS 2024. After application of survey weights, the corresponding population estimates were 50.5% (n=997) for HINTS 2022 and 49.5% (n=976) for HINTS 2024. The weighted mean age of respondents was 65.8 (95% CI 64.5-67.1) years. Approximately 52.1% (95% CI 48.4%-55.6%) of respondents were female survivors. The sample was predominantly non-Hispanic White (77.8%, 95% CI 74.6%-80.7%) survivors, although Hispanic (8.4%, 95% CI 6.7%-10.5%), non-Hispanic Black (7.3%, 95% CI 5.7%-9.2%), non-Hispanic Asian (2.0%, 95% CI 1.3%-2.9%), and non-Hispanic Other (4.6%, 95% CI 3.1%-6.8%) populations were also represented. Educational attainment varied substantially across respondents. Approximately 42.3% (95% CI 39.2%-45.6%) reported some college education, while 16.9% (95% CI 14.8%-19.2%) reported a bachelor’s degree and 16.8% (95% CI 14.7%-19.3%) reported postbaccalaureate education. Approximately 6.0% (95% CI 4.5%-7.8%) of respondents reported less than a high school education. Household income levels also varied across the sample. Approximately 22.0% (95% CI 18.7%-25.7%) of respondents reported annual household incomes between US $50,000 and US $74,999, while 21.6% (95% CI 18.6%-25.0%) reported incomes of at least US $100,000 annually. Approximately 16.3% (95% CI 13.8%-19.3%) reported annual household incomes below US $20,000. Of the 1972 cancer survivors included in the pooled dataset, between 1366 and 1373 respondents provided valid responses to the individual cancer information burden items. The composite cancer information burden score was available for 1389 respondents because it was calculated as the mean of available responses across the 4 burden items. After complete-case exclusion of respondents with missing covariate information, primarily household income, the final regression samples ranged from 952 to 957 participants. The composite burden analysis included 969 respondents.

**Figure 1.**
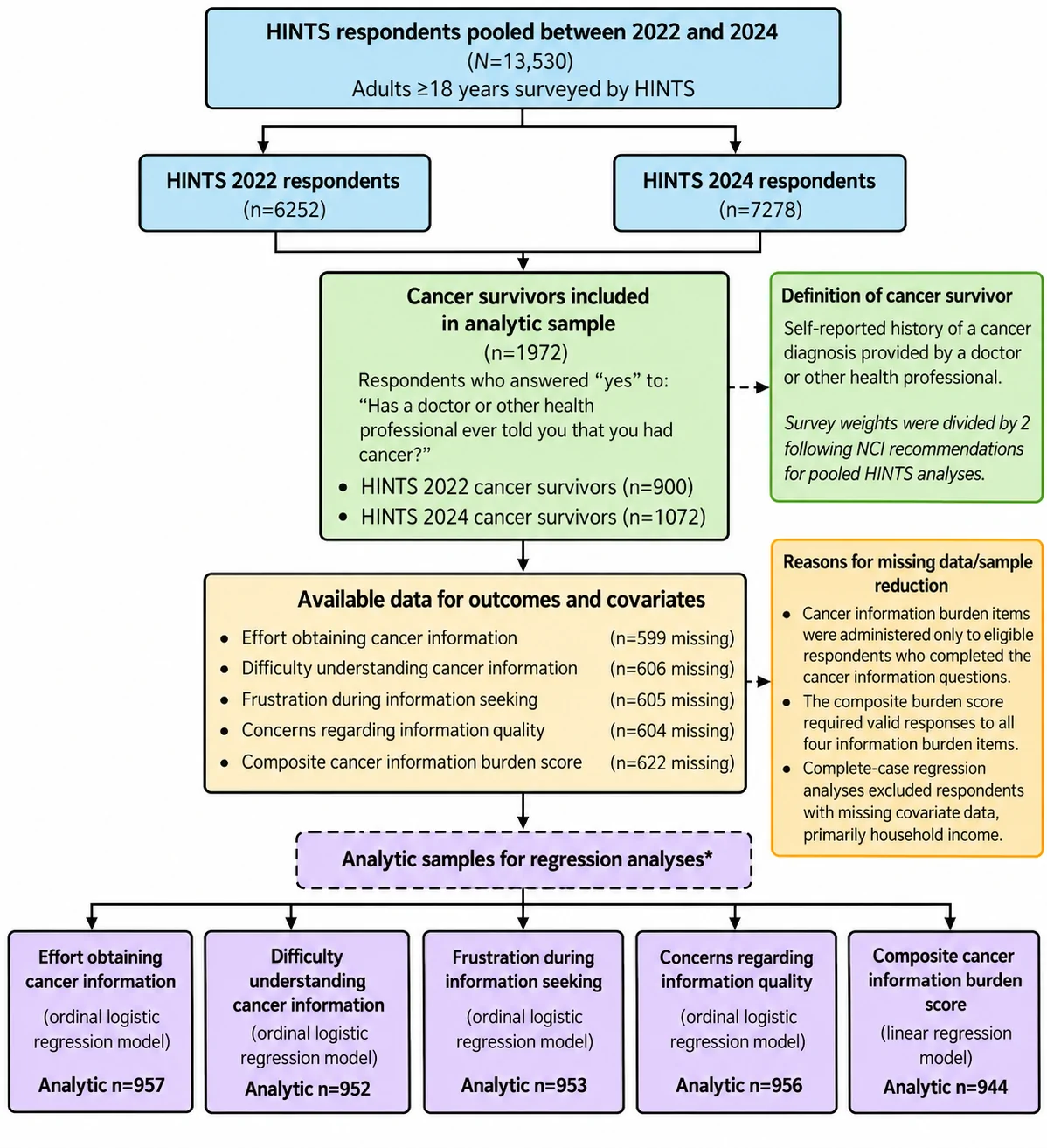
Study population selection and analytic sample flowchart. Health Information National Trends Survey (HINTS) 2022 and HINTS 2024 were the most recent publicly available survey cycles. No standard HINTS survey was conducted in 2023. N and n represent unweighted sample sizes. The composite cancer information burden score was calculated only for respondents with valid responses to all 4 burden items (n=1350). *Analytic n represents the complete-case sample used for each primary regression model after excluding respondents with missing covariate data. NCI: National Cancer Institute.

**Table 1.** Weighted descriptive characteristics of US cancer survivors included in the analytic sample^a^.

Characteristic	Unweighted, n	Weighted
Age (y), mean (95% CI)	1934	65.8 (64.5-67.1)
Composite cancer information burden score, mean (95% CI)	1389	1.24 (1.17-1.31)
Sex, % (95% CI)		
Female	1118	52.1 (48.4-55.6)
Male	828	47.9 (44.4-51.6)
Race and ethnicity, % (95% CI)		
Non-Hispanic White	1338	77.8 (74.6-80.7)
Non-Hispanic Black	207	7.3 (5.7-9.2)
Hispanic	184	8.4 (6.7-10.5)
Non-Hispanic Asian	44	2.0 (1.3-2.9)
Non-Hispanic Other	58	4.6 (3.1-6.8)
Educational attainment, % (95% CI)		
Less than high school	117	6.0 (4.5-7.8)
High school graduate	315	18.0 (15.6-20.6)
Some college	610	42.3 (39.2-45.6)
Bachelor’s degree	476	16.9 (14.8-19.2)
Postbaccalaureate degree	427	16.8 (14.7-19.3)
Household income (US $), % (95% CI)		
Less than 20,000	308	16.3 (13.8-19.3)
20,000-34,999	245	17.6 (13.9-22.0)
35,000-49,999	241	16.4 (13.8-19.3)
50,000-74,999	294	22.0 (18.7-25.7)
75,000-99,999	115	6.1 (4.5-8.1)
100,000 or greater	284	21.6 (18.6-25.0)
Survey cycle, % (95% CI)		
HINTS^b^ 2022	900	50.5 (47.3-53.6)
HINTS 2024	1072	49.5 (46.4-52.7)
Effort in obtaining cancer information, % (95% CI)		
Strongly disagree	355	25.2 (21.7-28.9)
Somewhat disagree	470	35.1 (31.4-38.9)
Somewhat agree	411	30.4 (26.6-34.5)
Strongly agree	137	9.4 (7.2-12.2)
Cancer information is hard to understand, % (95% CI)		
Strongly disagree	414	30.2 (26.4-34.3)
Somewhat disagree	487	34.8 (31.1-38.7)
Somewhat agree	367	28.1 (24.3-32.1)
Strongly agree	98	6.9 (5.2-9.2)
Cancer information seeking is frustrating, % (95% CI)		
Strongly disagree	446	31.5 (27.8-35.4)
Somewhat disagree	443	32.5 (29.0-36.2)
Somewhat agree	355	27.8 (24.4-31.5)
Strongly agree	123	8.2 (6.3-10.6)
Concerned about cancer information quality, % (95% CI)		
Strongly disagree	323	22.3 (19.1-25.8)
Somewhat disagree	365	26.9 (23.5-30.7)
Somewhat agree	451	35.9 (32.3-39.7)
Strongly agree	229	14.9 (12.2-18.0)

a Unweighted counts are presented alongside survey-weighted percentages or means and their 95% CIs. Survey-weighted estimates account for the complex HINTS sampling design. Percentages may not sum to 100% because of rounding.

b HINTS: Health Information National Trends Survey.

Regarding measures of cancer information burden, approximately 39.8% of respondents somewhat or strongly agreed that obtaining cancer information required substantial effort. Similarly, 35.0% somewhat or strongly agreed that cancer information was difficult to understand, while 36.0% somewhat or strongly agreed that seeking cancer information was frustrating. Concerns regarding cancer information quality were also common, with approximately 50.8% somewhat or strongly agreeing that they were concerned about the quality of cancer-related information. The 4 cancer information burden items demonstrated high internal consistency (Cronbach α=0.881). Exploratory factor analysis identified 1 dominant factor with an eigenvalue of 2.948, accounting for 73.7% of the total variance. All 4 items loaded strongly on this factor, with loadings ranging from 0.820 to 0.898. These findings supported the use of the mean of the 4 items as an overall cancer information burden score.

### Factors Associated With Cancer Information Burden

Parallel-lines diagnostics indicated evidence of nonproportionality for perceived effort in obtaining cancer information and concern regarding information quality. Diagnostic findings for difficulty understanding cancer information were mixed, whereas no evidence of nonproportionality was identified for frustration during information seeking. Partial proportional-odds sensitivity models were therefore estimated for outcomes showing evidence of assumption violations. These sensitivity analyses produced conclusions that were substantively consistent with those of the primary proportional-odds models. Table 2 presents survey-weighted regression models examining factors associated with cancer information burden among US cancer survivors. Several significant demographic and socioeconomic differences emerged across perceived effort in obtaining cancer information, difficulty understanding cancer information, frustration during information seeking, concern regarding information quality, and the composite cancer information burden score.

Hispanic cancer survivors experienced greater cancer information burden than non-Hispanic White survivors for selected outcomes. Hispanic respondents had significantly higher odds of reporting greater effort in obtaining cancer information (OR 2.611, 95% CI 1.387-4.913, *P=*.003) and greater frustration during cancer information seeking (OR 2.087, 95% CI 1.154-3.773, *P=*.02). Hispanic survivors also had higher composite cancer information burden scores, although this association did not reach statistical significance after adjustment for covariates (β=0.265, 95% CI −0.002 to 0.532, *P*=.051). These findings indicate that Hispanic cancer survivors reported greater cancer information burden for selected outcomes. Because the data are cross-sectional, the observed associations do not establish why these differences occurred.

**Table 2.** Survey-weighted ordinal logistic and linear regression models of cancer information burden among US cancer survivors^a^.

Variable	Effort in obtaining cancer information, OR^b^ (95% CI)	Difficulty understanding cancer information, OR (95% CI)	Frustration during information seeking, OR (95% CI)	Concern regarding information quality, OR (95% CI)	Composite cancer information burden, β^c^ (95% CI)
Age (y)	0.993 (0.977-1.010)	0.988 (0.973-1.004)	0.991 (0.977-1.006)	0.986 (0.971-1.001)	−0.005 (−0.011 to 0.002)
Sex (reference: male)					
Female	0.782 (0.554-1.106)	0.562 (0.383-0.824)^d^	0.683 (0.489-0.955)^e^	0.747 (0.539-1.034)	−0.177 (−0.313 to −0.041)^e^
Race and ethnicity (reference: non-Hispanic White)					
Non-Hispanic Black	1.381 (0.565-3.376)	0.809 (0.359-1.826)	0.811 (0.357-1.843)	1.153 (0.544-2.444)	0.047 (−0.257 to 0.351)
Hispanic	2.611 (1.387-4.914)^d^	1.681 (0.769-3.671)	2.087 (1.154-3.774)^e^	1.066 (0.573-1.981)	0.265 (−0.002 to 0.532)
Non-Hispanic Asian	5.495 (1.542-19.584)^d^	1.577 (0.385-6.465)	2.898 (0.687-12.230)	1.363 (0.367-5.062)	0.480 (−0.083 to 1.044)
Non-Hispanic other	2.974 (0.806-10.980)	0.392 (0.119-1.293)	1.324 (0.458-3.823)	2.798 (1.223-6.401)^e^	0.176 (−0.085 to 0.436)
Educational attainment (reference: less than high school)					
High school graduate	0.495 (0.132-1.852)	0.766 (0.223-2.630)	0.334 (0.088-1.268)	0.376 (0.090-1.568)	−0.338 (−0.824 to 0.149)
Some college	0.868 (0.250-3.017)	0.774 (0.244-2.461)	0.344 (0.095-1.247)	0.335 (0.082-1.372)	−0.306 (−0.767 to 0.155)
Bachelor’s degree	0.591 (0.158-2.202)	0.480 (0.152-1.518)	0.270 (0.072-1.019)	0.438 (0.105-1.825)	−0.399 (−0.875 to 0.077)
Postbaccalaureate degree	0.594 (0.162-2.172)	0.506 (0.156-1.640)	0.256 (0.068-0.955)^e^	0.354 (0.084-1.493)	−0.440 (−0.919 to 0.038)
Household income (reference: less than US $20,000)					
20,000-34,999	0.774 (0.306-1.953)	0.791 (0.306-2.047)	0.518 (0.219-1.228)	0.865 (0.357-2.094)	−0.163 (−0.506 to 0.181)
35,000-49,999	0.675 (0.289-1.579)	1.713 (0.737-3.978)	0.933 (0.426-2.042)	1.323 (0.576-3.038)	0.039 (−0.279 to 0.357)
50,000-74,999	0.711 (0.318-1.589)	1.065 (0.480-2.366)	0.901 (0.436-1.862)	1.159 (0.518-2.595)	−0.023 (−0.321 to 0.276)
75,000-99,999	0.561 (0.179-1.760)	1.956 (0.610-6.273)	1.480 (0.517-4.231)	1.566 (0.544-4.510)	0.092 (−0.349 to 0.532)
100,000 or greater	0.507 (0.204-1.262)	0.855 (0.369-1.978)	0.819 (0.358-1.875)	1.025 (0.427-2.460)	−0.151 (−0.491 to 0.188)
Survey cycle (reference: HINTS^f^ 2022)					
HINTS 2024	1.002 (0.625-1.604)	1.086 (0.671-1.756)	0.875 (0.550-1.392)	0.697 (0.451-1.077)	−0.045 (−0.222 to 0.131)
Observations, n	957	952	953	956	969
Overall model, *P* value	<.001	.006	.04	.40	.009
*R*²	N/A^g^	N/A	N/A	N/A	.068

a ORs greater than 1 indicate higher odds of reporting a higher level of cancer information burden. The proportional-odds assumption was evaluated using parallel-lines tests, and partial proportional-odds sensitivity models were estimated for outcomes showing evidence of nonproportionality.

b OR: odds ratio.

c β: unstandardized linear regression coefficient.

d *P*<.01.

e *P*<.05.

f HINTS: Health Information National Trends Survey.

g N/A: not applicable.

Non-Hispanic Asian cancer survivors also demonstrated elevated cancer information burden for selected outcomes. In particular, Asian survivors had significantly higher odds of reporting greater effort in obtaining cancer information than non-Hispanic White survivors (OR 5.495, 95% CI 1.543-19.568, *P=*.009). Estimates for frustration and the composite burden score were higher, but these associations did not reach statistical significance. The wide CIs likely reflect the smaller number of non-Hispanic Asian respondents in the analytic sample.

Female cancer survivors generally reported a lower cancer information burden than male survivors. Female survivors had significantly lower odds of reporting difficulty understanding cancer information (OR 0.562, 95% CI 0.383-0.824, *P=*.003) and greater frustration when seeking cancer information (OR 0.683, 95% CI 0.489-0.954, *P=*.03). They also had significantly lower overall cancer information burden scores (β=−0.177, 95% CI −0.313 to −0.041, *P*=.01). These associations may be related to differences in health information-seeking experience or familiarity with health care navigation, although these potential explanations were not directly measured in HINTS.

Educational attainment was associated with frustration during cancer information seeking. Respondents with postbaccalaureate education had significantly lower odds of reporting greater frustration than respondents with less than a high school education (OR 0.256, 95% CI 0.069-0.951, *P=*.042). Respondents with a bachelor’s degree showed a similar pattern, although the association narrowly missed statistical significance (OR 0.270, 95% CI 0.072-1.016, *P=*.053). Other education-related estimates generally suggested lower cancer information burden among respondents with higher educational attainment, but the CIs included the null value.

Non-Hispanic respondents classified in the Other racial and ethnic category had significantly higher odds of reporting greater concern regarding cancer information quality than non-Hispanic White respondents (OR 2.798, 95% CI 1.222-6.408, *P=*.02). This estimate should be interpreted cautiously because of the heterogeneity and likely small sample size of this category.

Age was not significantly associated with the cancer information burden outcomes after covariate adjustment. Household income and US census division also demonstrated limited associations across the models. Differences between the 2022 and 2024 HINTS cycles were not statistically significant for any outcome. These findings indicate that the measured associations did not differ significantly between the 2 survey cycles.

Figure 2 presents exploratory, survey-weighted mean cancer information burden scores across US census divisions. Certain divisions appeared to have higher or lower mean scores for selected burden measures, but the patterns were not uniform across outcomes or survey cycles. No formal pairwise or omnibus comparisons were conducted for this figure; therefore, these descriptive differences should not be interpreted as statistically significant geographic variation. Because the number and weighted distribution of cancer survivors differed across census divisions and survey cycles, some of the visual variation may reflect differences in regional sample composition. Multimedia Appendix 1 reports the unweighted sample sizes and weighted percentages of cancer survivors by census division and survey cycle.

The regional distribution of cancer survivors did not differ significantly between HINTS 2022 and HINTS 2024, based on a survey-weighted design-adjusted Pearson test (*F*_7.48,2594.52_=0.943, *P*=.48). Thus, the descriptive differences shown in Figure 2 were not accompanied by evidence of a significant change in the geographic composition of the cancer-survivor sample across survey cycles.

**Figure 2.**
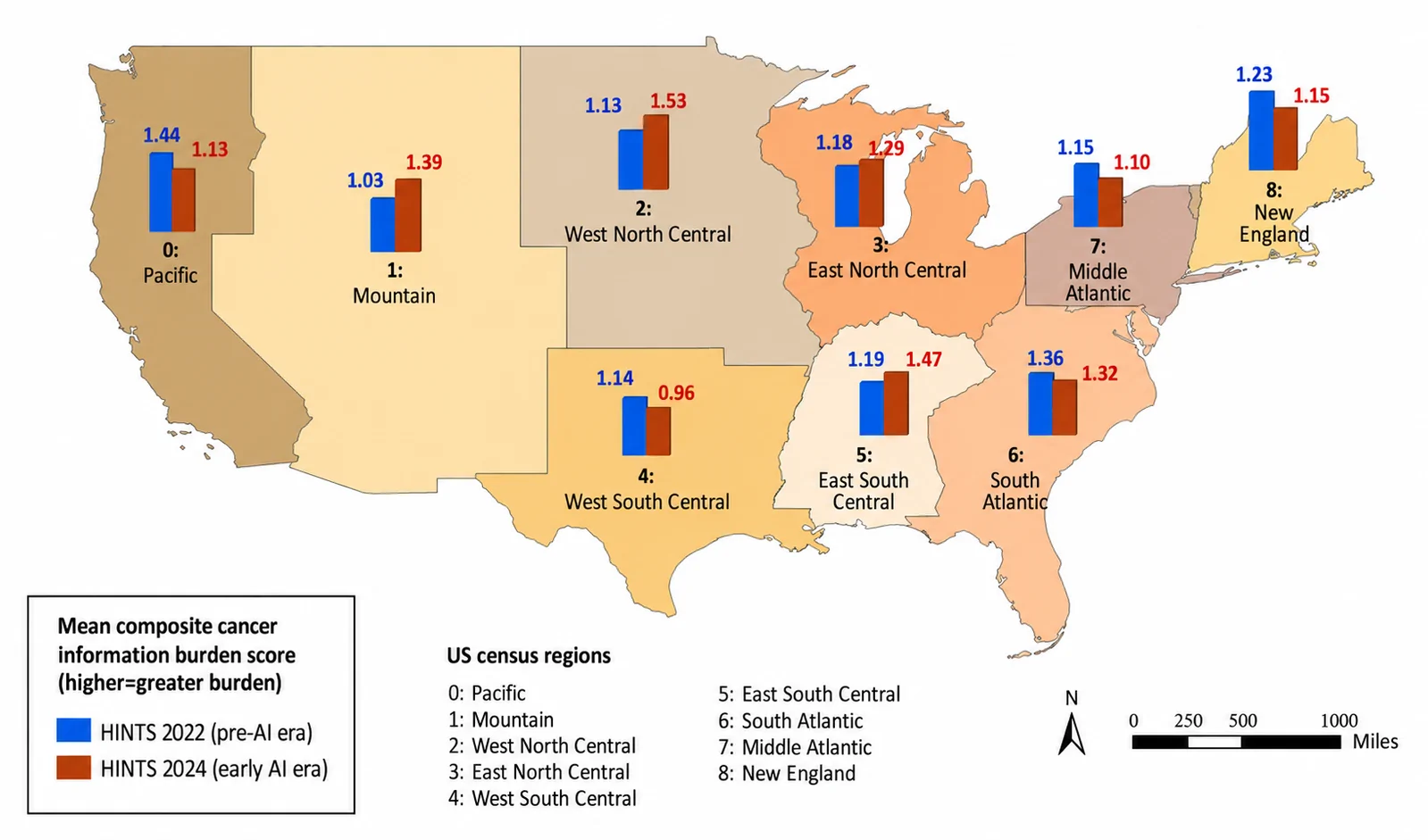
Exploratory, survey-weighted mean cancer information burden scores across US census regions (Health Information National Trends Survey [HINTS] 2022 vs HINTS 2024). Values represent descriptive survey-weighted means for perceived effort in obtaining cancer information, difficulty understanding cancer information, frustration during information seeking, concern regarding information quality, and the composite burden score across the US census divisions. No formal pairwise or omnibus statistical comparisons were conducted. Regional patterns should therefore be interpreted as exploratory. Unweighted sample sizes and weighted distributions by census division and survey cycle are reported in Multimedia Appendix 1. Data source: HINTS 2022 and 2024 survey-weighted means estimated using linearized variance.

### Secondary Analysis of the Interaction Between Age and Survey Cycle

Table 3 presents the secondary interaction analyses examining whether age-related associations with cancer information burden differed between HINTS 2022 and HINTS 2024. Across all 5 outcomes, the age × survey-cycle interaction terms were not statistically significant, indicating no evidence that the association between age and cancer information burden differed between HINTS 2022 and HINTS 2024. For effort in obtaining cancer information, the interaction between age and HINTS 2024 was not statistically significant (β=−0.004, 95% CI −0.034 to 0.025, *P*=.77). Similarly, no significant age-by-cycle interaction was observed for difficulty understanding cancer information (β=−0.019, 95% CI −0.058 to 0.020, *P*=.33), frustration during cancer information seeking (β=−0.012, 95% CI −0.043 to 0.020, *P*=.47), concerns regarding cancer information quality (β=−0.012, 95% CI −0.047 to 0.024, *P*=.52), or the composite cancer information burden score (β=−0.005, 95% CI −0.018 to 0.008, *P*=.45).

**Table 3.** Interaction between age and survey year in models of cancer information burden among US cancer survivors^a^.

Outcome	Age, β (95% CI)	HINTS^b^ 2024, β (95% CI)	Age × HINTS 2024, β (95% CI)	*P* value	Observations
Effort in obtaining cancer information	−0.003 (−0.025 to 0.018)	0.296 (−1.811 to 2.402)	−0.004 (−0.034 to 0.025)	.77	957
Difficulty understanding cancer information	0.003 (−0.030 to 0.035)	1.364 (−1.376 to 4.104)	−0.019 (−0.058 to 0.020)	.33	952
Frustration during information seeking	0.000 (−0.026 to 0.026)	0.644 (−1.596 to 2.885)	−0.012 (−0.043 to 0.020)	.47	953
Concerns regarding information quality	−0.006 (−0.036 to 0.025)	0.422 (−2.165 to 3.009)	−0.012 (−0.047 to 0.024)	.52	956
Composite cancer information burden score	−0.001 (−0.011 to 0.010)	0.288 (−0.613 to 1.188)	−0.005 (−0.018 to 0.008)	.45	969

a Survey-weighted proportional-odds ordinal logistic regression models were estimated for the 4 ordinal cancer information burden outcomes. For these models, β estimates are unexponentiated coefficients expressed on the log-odds scale. Survey-weighted linear regression was used for the composite cancer information burden score, for which β estimates are unstandardized linear regression coefficients. Models were adjusted for sex, race and ethnicity, educational attainment, household income, and US census division. The age × survey-cycle interaction term indicates whether the association between age and each outcome differed between HINTS 2022 and HINTS 2024. HINTS 2022 was the reference survey cycle.

b HINTS: Health Information National Trends Survey.

Figure 3 presents predictive margins that illustrate age-specific trends in cancer information burden across the 2022 and 2024 survey cycles. Overall, predicted probabilities and burden scores were broadly comparable between survey years, with overlapping CIs across most age groups. Across several outcomes, younger cancer survivors demonstrated modestly higher predicted levels of cancer information burden compared with older survivors, particularly in 2024. This pattern was most apparent for difficulty understanding cancer information, concerns regarding cancer information quality, and the composite cancer information burden score. However, CIs were wider at younger ages, indicating greater uncertainty in these estimates. Collectively, these findings indicate that age-related associations with cancer information burden did not differ significantly between the 2022 and 2024 survey cycles. Because HINTS did not measure GenAI use or exposure, these findings cannot be attributed to changes in AI-enabled health information environments.

**Figure 3.**
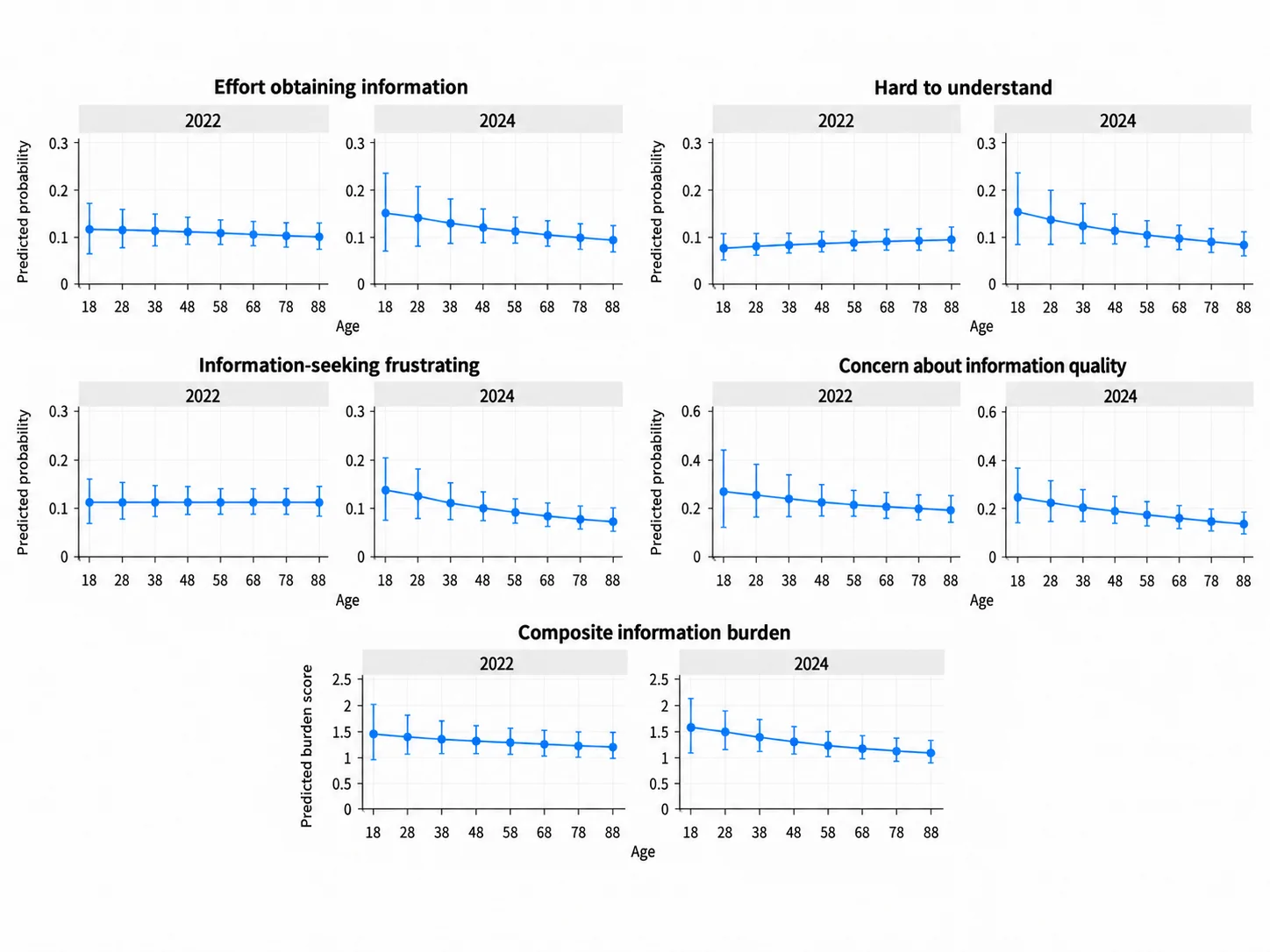
Predicted cancer information burden by age and survey year among US cancer survivors.

## Discussion

### Principal Findings and Interpretation

This study provides an updated, nationally representative assessment of cancer information-seeking burden among US cancer survivors using HINTS 2022 and 2024 data. The findings were broadly consistent with earlier HINTS-based and population-based studies showing that cancer information-seeking behaviors and experiences differ across demographic and socioeconomic groups [15-18]. The present analysis extends this literature by examining 4 burden dimensions separately, evaluating a 4-item composite measure, and assessing whether these associations were evident in recent HINTS cycles. Accordingly, the findings should be interpreted primarily as a contemporary and confirmatory update rather than as evidence of entirely new demographic patterns. In contrast, adjusted differences between the 2022 and 2024 survey cycles and age × survey-cycle interactions were not statistically significant. These findings should be interpreted as evidence regarding cancer information burden during 2022 and 2024 and not as evidence of GenAI effects because HINTS did not assess AI use, awareness, or exposure. Viewed through the Comprehensive Model of Information Seeking, the observed associations may reflect differences in antecedent characteristics that shape information-seeking experiences. However, the current study did not measure several constructs central to the framework, including the salience of current information needs, beliefs, prior cancer experience, information-channel characteristics, trust, digital access, and eHealth literacy. The findings therefore support a theory-informed interpretation but should not be considered a formal test of the model. The secondary age × survey-cycle analyses did not identify statistically significant interactions for any outcome. These results indicate that the association between age and cancer information burden did not differ significantly across the 2 survey cycles, although the analyses may have had limited power to detect modest interaction effects.

One of the most notable findings was the elevated cancer information burden reported among Hispanic cancer survivors. Hispanic respondents consistently demonstrated higher perceived effort associated with obtaining cancer information and greater frustration during cancer information seeking compared with non-Hispanic White survivors. Non-Hispanic Asian respondents also demonstrated significantly higher perceived effort in obtaining cancer information. These findings are consistent with prior research documenting differences in health literacy, access to culturally tailored information, and digital navigation experiences across population groups [20,26-28]. The associations remained after adjustment for education, income, and geographic region. However, the present study did not directly measure health literacy, structural barriers, digital navigation skills, or exposure to AI-generated health content. These factors therefore represent possible areas for future investigation rather than established explanations for the observed associations. Overall, these findings are consistent with prior HINTS-based and population-based research. Davis et al [15] identified demographic correlates of cancer information-seeking experiences among US cancer survivors, while Elkefi and Matthews [16] documented differences in information-seeking behaviors and source preferences across a diverse survivor sample. Mahmood et al [17] further showed that patient-centered communication was associated with cancer information-seeking experiences, and Benzo et al [18] reported associations between digital information-seeking behaviors and sociodemographic characteristics, cancer history, and perceived health. The present study extends this literature by showing that selected demographic differences remain evident in recent HINTS data and vary across specific dimensions of cancer information-seeking burden.

The descriptive patterns shown in Figure 2 suggest possible geographic variation in cancer information burden, but these observations are exploratory. The figure was not accompanied by formal pairwise or omnibus testing, and the number and weighted distribution of cancer survivors varied across census divisions and survey cycles. Consequently, some visual differences may reflect regional sample composition rather than independent geographic effects. Census division was included as a covariate in the adjusted regression models, but most division-specific estimates were not statistically significant. Future studies with larger geographic samples should formally test regional differences and examine whether survey-cycle changes vary across areas.

Educational attainment also demonstrated a protective relationship with several dimensions of cancer information burden. Cancer survivors with bachelor’s or postbaccalaureate degrees reported lower levels of frustration during cancer information seeking and generally lower composite burden scores. These associations may be related to differences in digital literacy, familiarity with health systems, or the ability to evaluate complex health information. However, these potential mechanisms were not directly measured in the present study. Prior research has consistently demonstrated that health literacy and digital literacy strongly influence the ability to effectively navigate online health information environments [29,30]. Earlier HINTS-based studies have also linked educational attainment and socioeconomic characteristics with cancer information-seeking experiences and source use [15,17]. The current findings therefore reinforce prior evidence that educational resources may shape how survivors experience the effort and frustration associated with obtaining cancer information. The present findings indicate that educational attainment was associated with selected dimensions of perceived cancer information burden. The study cannot determine whether this association was influenced by the increasing availability of AI-enabled information tools.

Interestingly, female cancer survivors reported significantly less difficulty understanding cancer information and less frustration during information seeking than male survivors. Prior HINTS-based and population-based studies have also identified sex differences in health information-seeking behaviors, preventive service use, and engagement with health care systems [31,32]. Female survivors may have greater prior experience seeking health information, communicating with health professionals, and managing health care for themselves or family members. These experiences may provide context for the observed associations. However, the present study did not directly measure prior information-seeking frequency, caregiving roles, digital literacy, or health care navigation experience, and these factors should not be interpreted as established explanatory mechanisms. Future studies should directly evaluate whether these characteristics account for sex differences in cancer information-seeking burden.

Age was examined as a potential modifier because digital access, online information-seeking experience, and familiarity with digital health tools may differ across age groups. However, the absence of statistically significant interactions suggests that age-related patterns were not detectably different between the 2022 and 2024 cycles. Because HINTS did not directly measure digital literacy, technology use, or AI exposure, the mechanisms underlying age-related information experiences could not be evaluated. Future studies with larger samples and direct measures of digital engagement should further assess whether age modifies the relationship between changing information environments and cancer information burden.

Although AI was not evaluated in this study, the findings provide a baseline for future research on cancer communication in changing digital environments. GenAI tools may offer opportunities to simplify medical terminology, tailor educational materials, and support patient engagement, but they may also introduce concerns related to information accuracy, bias, and unequal access. Because HINTS did not measure AI use, exposure, or reliance on AI-generated information, the present results cannot determine whether AI-enabled systems influenced cancer information burden. Future studies should directly assess the use of these tools and evaluate their associations with comprehension, trust, and information burden.

The incremental contribution of this study lies in its use of recent pooled HINTS cycles, its simultaneous evaluation of 4 specific burden dimensions, and its psychometric assessment of a composite cancer information burden measure. The high internal consistency and single-factor structure supported the use of the composite, while the item-specific models showed that associations were not uniform across all dimensions. This outcome-specific pattern provides more detail than treating cancer information seeking as a single behavior or experience.

### Limitations

This study has several limitations. First, the cross-sectional design precludes causal inference. Second, the study relied on self-reported perceptions of cancer information burden rather than objective measures of information-seeking behavior or comprehension. Third, the analyses did not account for cancer-specific characteristics, including cancer type, time since diagnosis, treatment status, disease stage or severity, recurrence, or survivorship phase. These factors may influence information-seeking needs and perceived burden and could contribute to residual confounding. Future studies should examine whether the observed associations vary across cancer types and phases of care. The analyses also did not adjust for factors more directly related to digital information access and navigation, such as internet use, frequency of online health information seeking, digital literacy, eHealth literacy, or access to digital technologies. These factors may influence perceived cancer information burden and could partly explain the observed associations. Future studies should incorporate these measures to better understand the pathways through which demographic and socioeconomic characteristics are associated with cancer information burden. Fourth, HINTS 2022 and 2024 did not measure GenAI awareness, use, chatbot engagement, AI-assisted search behavior, or exposure to AI-generated health information. Survey cycle therefore cannot be interpreted as a proxy for AI exposure, and neither differences nor the absence of differences between 2022 and 2024 can be attributed to GenAI. References to changes in the digital information environment are intended only to provide broader temporal context. Fifth, some subgroup analyses, particularly among non-Hispanic Asian respondents, were limited by relatively small sample sizes, resulting in wider CIs. Finally, although the study examined several dimensions of cancer information burden, additional factors such as trust in online information, patient portal engagement, and exposure to AI-generated misinformation warrant further investigation. Complete-case analysis reduced the adjusted analytic samples, primarily because of missing household income data. If missingness was systematically related to information burden, some selection bias may remain.

Despite these limitations, this study provides nationally representative evidence regarding cancer information burden in 2022 and 2024. The findings identify demographic and socioeconomic characteristics associated with selected dimensions of information burden during this period. Because the survey did not measure AI-related exposures, the study cannot determine whether technological developments influenced these associations or whether AI-enabled communication systems affected information burden. Future research should directly measure AI tool use and examine its longitudinal associations with patient understanding, trust, well-being, and cancer information burden.

### Implications for Cancer and Digital Health Communication

Digital health information systems may create opportunities to improve access to understandable and personalized cancer information. However, their effects on cancer information burden were not evaluated in this study. The observed associations by race and ethnicity, sex, and educational attainment underscore the importance of accessibility, cultural responsiveness, readability, and health literacy–sensitive design in cancer communication. Future evaluations of AI-enabled communication tools should examine whether their benefits and limitations differ across population groups.

### Conclusions

These findings reinforce and update prior HINTS-based evidence that cancer information-seeking experiences differ across survivor populations while showing that associations vary by the specific dimension of burden examined. The validated composite measure may support future studies seeking to monitor cancer information burden over time or across communication environments. Hispanic and non-Hispanic Asian survivors, and individuals with lower educational attainment, may experience greater difficulty obtaining, understanding, and evaluating cancer information. Cancer communication strategies should prioritize accessibility, readability, cultural responsiveness, and health literacy. Future studies should directly measure the use of GenAI and other emerging digital tools before evaluating their relationship with cancer information burden.

## Supplementary material

10.2196/101310 Multimedia Appendix 1 Distribution of cancer survivors by US census division and Health Information National Trends Survey (HINTS) cycle.

## Abbreviations

GenAI: generative AI
HINTS: Health Information National Trends Survey
OR: odds ratio

## References

[1] Shaffer KM, Turner KL, Siwik C (2023). Digital health and telehealth in cancer care: a scoping review of reviews. Lancet Digit Health.

[2] Lazarou I, Krooupa AM, Nikolopoulos S (2024). Cancer patients’ perspectives and requirements of digital health technologies: a scoping literature review. Cancers (Basel).

[3] Briggs LG, Labban M, Alkhatib K, Nguyen DD, Cole AP, Trinh QD (2022). Digital technologies in cancer care: a review from the clinician’s perspective. J Comp Eff Res.

[4] Yun HS, Bickmore T (2025). Online health information–seeking in the era of large language models: cross-sectional web-based survey study. J Med Internet Res.

[5] Wardle C, Urbani S, Wang E (2025). Evolving health information–seeking behavior in the context of Google AI Overviews, ChatGPT, and Alexa: interview study using the think-aloud protocol. J Med Internet Res.

[6] Budenz A, Sleight AG, Klein WMP (2022). A qualitative study of online information-seeking preferences among cancer survivors. J Cancer Surviv.

[7] Miller LE (2014). Uncertainty management and information seeking in cancer survivorship. Health Commun.

[8] Ferraris G, Monzani D, Coppini V (2023). Barriers to and facilitators of online health information-seeking behaviours among cancer patients: a systematic review. Digit Health.

[9] Wu Y, Zhang L, Zhao X (2025). Linking online health information seeking to cancer information overload among Chinese cancer patients’ family members. Digit Health.

[10] Peng W, Carcioppolo N, Occa A, Ali K, Yang Q, Yang F (2021). Feel worried, overloaded, or fatalistic? The determinants of cancer uncertainty management preferences. Health Commun.

[11] Watson A, van Kessel K (2018). Cancer survivors’ experiences and explanations of postcancer fatigue: an analysis of online blogs. Cancer Nurs.

[12] Sørensen K (2024). Fostering digital health literacy to enhance trust and improve health outcomes. Comput Methods Programs Biomed Update.

[13] Papp-Zipernovszky O, Horváth MD, Schulz PJ, Csabai M (2021). Generation gaps in digital health literacy and their impact on health information seeking behavior and health empowerment in Hungary. Front Public Health.

[14] Mackert M, Mabry-Flynn A, Champlin S, Donovan EE, Pounders K (2016). Health literacy and health information technology adoption: the potential for a new digital divide. J Med Internet Res.

[15] Davis SN, O’Malley DM, Bator A, Ohman-Strickland P, Hudson SV (2021). Correlates of information seeking behaviors and experiences among adult cancer survivors in the USA. J Cancer Educ.

[16] Elkefi S, Matthews AK (2024). Exploring health information-seeking behavior and information source preferences among a diverse sample of cancer survivors: implications for patient education. J Cancer Educ.

[17] Mahmood A, Kedia S, Ogunsanmi DO, Kabir U, Entwistle C (2025). Patient-centered communication and cancer information-seeking experiences among cancer survivors: a population-based study in the United States. Patient Educ Couns.

[18] Benzo RM, Tetrick MK, Krok-Schoen JL (2025). Digital information-seeking behaviors among cancer survivors: associations with sociodemographic determinants, cancer history, and perceived health. J Cancer Surviv.

[19] Gordon NP, Hornbrook MC (2018). Older adults’ readiness to engage with eHealth patient education and self-care resources: a cross-sectional survey. BMC Health Serv Res.

[20] Valdez RS, Rogers CC (2022). Consumer health informatics for racial and ethnic minoritized communities: minor progress, major opportunities. Yearb Med Inform.

[21] Rousseau J, Gibbs L, Garcia-Cabrera C (2024). A pioneering EMR-embedded digital health literacy tool reveals healthcare disparities for diverse older adults. J Am Geriatr Soc.

[22] Johnson JD, Donohue WA, Atkin CK, Johnson S (1995). A comprehensive model of information seeking: tests focusing on a technical organization. Sci Commun.

[23] Ruppel EK (2016). Scanning health information sources: applying and extending the comprehensive model of information seeking. J Health Commun.

[24] Yang Q, Van Stee SK, Rains SA (2023). Comprehensive model of information seeking: a meta-analysis. J Health Commun.

[25] Reifegerste D, Blech S, Dechant P (2020). Understanding information seeking about the health of others: applying the comprehensive model of information seeking to proxy online health information seeking. J Health Commun.

[26] Chesser A, Burke A, Reyes J, Rohrberg T (2016). Navigating the digital divide: a systematic review of eHealth literacy in underserved populations in the United States. Inform Health Soc Care.

[27] López L, Green AR, Tan-McGrory A, King R, Betancourt JR (2011). Bridging the digital divide in health care: the role of health information technology in addressing racial and ethnic disparities. Jt Comm J Qual Patient Saf.

[28] Whitehead L, Talevski J, Fatehi F, Beauchamp A (2023). Barriers to and facilitators of digital health among culturally and linguistically diverse populations: qualitative systematic review. J Med Internet Res.

[29] Gorski P (2005). Education equity and the digital divide. AACE Rev.

[30] Afzal A, Khan S, Daud S, Ahmad Z, Butt A (2023). Addressing the digital divide: access and use of technology in education. J Soc Sci Rev.

[31] Vaidya V, Partha G, Karmakar M (2012). Gender differences in utilization of preventive care services in the United States. J Womens Health (Larchmt).

[32] Figueroa CA, Luo T, Aguilera A, Lyles CR (2021). The need for feminist intersectionality in digital health. Lancet Digit Health.

