# Supplementary material for: Cancer Information Burden Among US Cancer Survivors Using Health Information National Trends Survey 2022 and 2024: Cross-Sectional Study
Source: JMIR Cancer. 2026 Oct 28;12:e101310. doi: 10.2196/101310 (PMC13717679; doi:10.2196/101310)
Supplement: Supplementary file 1 [file cancer-v12-e101310-s001.docx]

**Supplementary Table S2.** Distribution of Cancer Survivors by U.S. Census Division and HINTS Survey Cycle

| **U.S. Census division** | **HINTS 2022, unweighted n** | **HINTS 2024, unweighted n** | **Weighted % in HINTS 2022** | **Weighted % in HINTS 2024** |
| --- | --- | --- | --- | --- |
| New England | 39 | 51 | 57.6 | 42.4 |
| Middle Atlantic | 81 | 127 | 48.5 | 51.5 |
| East North Central | 115 | 134 | 53.3 | 46.7 |
| West North Central | 46 | 58 | 47.3 | 52.7 |
| South Atlantic | 226 | 258 | 47.1 | 52.9 |
| East South Central | 46 | 64 | 45.8 | 54.2 |
| West South Central | 142 | 146 | 57.6 | 42.4 |
| Mountain | 75 | 77 | 58.1 | 42.0 |
| Pacific | 130 | 157 | 45.8 | 54.2 |

**Note:** Weighted percentages are row percentages within each Census division. The survey-weighted design-adjusted Pearson test indicated no significant difference in regional distribution between HINTS 2022 and HINTS 2024, F(7.48, 2594.52)=0.943, P=.475.
